# Unravelling 2-oxoglutarate turnover and substrate oxidation dynamics in 5-methylcytosine-oxidising TET enzymes

**DOI:** 10.1038/s42004-024-01382-1

**Published:** 2024-12-20

**Authors:** Klemensas Šimelis, Roman Belle, Akane Kawamura

**Affiliations:** 1 Chemistry Research Laboratory, Department of Chemistry, University of Oxford, Oxford, UK; 2 Chemistry—School of Natural and Environmental Sciences, Bedson Building, Newcastle University, Newcastle upon Tyne, UK

**Keywords:** Enzyme mechanisms, Bioanalytical chemistry, Nucleic acids, Oxidoreductases

## Abstract

Fe(II)- and 2-oxoglutarate (2OG)-dependent dioxygenases use 2OG and O_2_ cofactors to catalyse substrate oxidation and yield oxidised product, succinate, and CO_2_. Simultaneous detection of substrate and cofactors is difficult, contributing to a poor understanding of the dynamics between substrate oxidation and 2OG decarboxylation activities. Here, we profile 5-methylcytosine (^5m^C)-oxidising Ten-Eleven Translocation (TET) enzymes using MS and ^1^H NMR spectroscopy methods and reveal a high degree of substrate oxidation-independent 2OG turnover under a range of conditions. 2OG decarboxylase activity is substantial (>20% 2OG turned over after 1 h) in the absence of substrate, while, under substrate-saturating conditions, half of total 2OG consumption is uncoupled from substrate oxidation. 2OG kinetics are affected by substrate and non-substrate DNA oligomers, and the sequence-agnostic effects are observed in amoeboflagellate *Naegleria gruberi* NgTet1 and human TET2. TET inhibitors also alter uncoupled 2OG kinetics, highlighting the potential effect of 2OG dioxygenase inhibitors on the intracellular balance of 2OG/succinate.

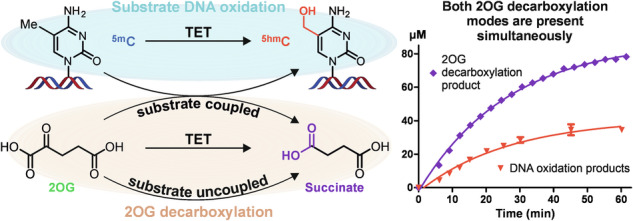

## Abstract (web summary)

The ten-eleven translocation (TET) dioxygenase subfamily catalyse the sequential oxidation of 5-methylcytosine (^5^^m^C) in DNA and belong to the Fe(II)-/2-oxoglutarate (2OG)-dependent dioxygenases that use 2OG and O_2_ cofactors to yield succinate and CO_2._ Here, the authors profile the TET-catalysed ^5^^m^C DNA oxidation and 2OG decarboxylation using MS and ^1^H NMR spectroscopy methods, revealing a high degree of substrate oxidation-independent 2OG turnover in TETs.

## Introduction

**Fig. 1:**
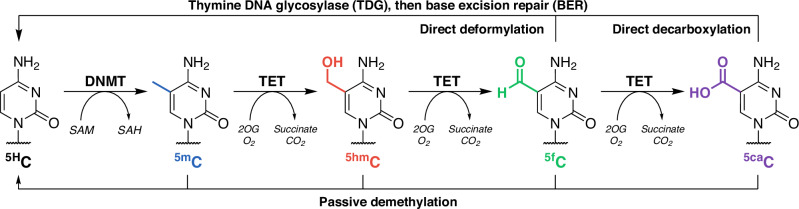
Pathways of dynamic cytosine modifications. Cytosine (^5H^C) is methylated by DNA methyltransferases (DNMTs) to produce ^5m^C, which can be iteratively oxidised to ^5hm^C, ^5f^C, and ^5ca^C by TETs in an Fe(II)/2OG-dependent manner^3^. ^5f^C and ^5ca^C can be replaced with ^5H^C via passive demethylation, TDG/BER mechanisms^4^, direct deformylation^46^, or decarboxylation^47,48^.

Fe(II)- and 2-oxoglutarate (2OG)-dependent dioxygenases constitute a diverse superfamily of enzymes involved in a wide variety of biological functions, including epigenetic regulation, collagen biosynthesis, and oxygen sensing^1^. The Ten-Eleven Translocation (TET) dioxygenase subfamily catalyses the sequential oxidation of 5-methylcytosine (^5m^C) to 5-hydroxymethylcytosine (^5hm^C), 5-formylcytosine (^5f^C), and 5-carboxycytosine (^5ca^C) using 2OG and O_2_ as co-substrates and releasing succinate and CO_2_ as by-products (Fig. 1)^2,3^.

Unmodified cytosine (^5H^C) can be regenerated by a combination of thymine DNA glycosylase (TDG) activity on bases ^5f^C and ^5ca^C followed by base excision repair (BER) mechanisms^4^. Cytosine methylation occurs predominantly on cytosine-guanine (CpG) dinucleotides, with up to 80% of genomic CpG sites methylated in mammals^5^. Correspondingly, TET DNA oxidation activity depends heavily on the sequence context of the modified cytosine (^5x^C) with a strong preference for ^5x^CpG dinucleotides, demonstrating a high degree of epigenetic synergy, although flanking nucleotides in positions −3 to +2 (relative to ^5x^C) also affect activity^6–8^. Furthermore, while CpG methylation at gene promoter regions is commonly associated with transcriptional silencing^9^, hydroxymethylation is predictive of poised chromatin^10^. In these ways, TETs play key epigenetic roles in developmental processes such as pre-implantation development^11^, genomic imprinting erasure^12^, and pluripotency regulation^13,14^. Accordingly, TET dysfunction is implicated in numerous oncological diseases. Decreased TET protein and ^5hm^C levels are associated with human breast, liver, lung, pancreatic, and prostate cancers^15^, while *TET2* loss-of-function mutations are prevalent in haematopoietic malignancies^16^. Substrate oxidation by Fe(II)/2OG-dependent dioxygenases is stoichiometrically linked to 2OG consumption. Oxidative decarboxylation of 2OG to succinate is a requisite for the formation of the reactive Fe(IV)-oxo complex^17^, but 2OG turnover can also take place in the absence of the associated substrate, resulting in uncoupled 2OG decarboxylation. Such activity has been observed in a number of 2OG dioxygenase subfamilies, including histone lysine demethylases (KDMs)^18^, prolyl/lysyl hydroxylases^19–21^, and DNA repair enzymes AlkB^22,23^, with uncoupled 2OG turnover rates reaching up to 10% of rates observed under substrate-saturating conditions. Uncoupled 2OG turnover has been observed in TET1^23^, but limited data is available on the interplay between TET DNA oxidation and 2OG decarboxylase activity, as to-date biochemical characterisation of TETs almost exclusively utilises MS^6,24–28^ or antibody-based^29–32^ methods for the detection of DNA substrate oxidation. Here, we describe the concurrent profiling of DNA substrate oxidation and 2OG decarboxylation by TETs using MS and ^1^H NMR spectroscopy methods. While substrate oxidation and 2OG decarboxylation were coupled as expected, we observed that TETs exhibited unusually high levels of uncoupled 2OG decarboxylation, which were reduced by 2OG competitive inhibitors, or enhanced in the presence of non-substrate oligonucleotides and substrate-competitive TET inhibitors.

## Results

### Characterisation of recombinant NgTet1

**Fig. 2:**
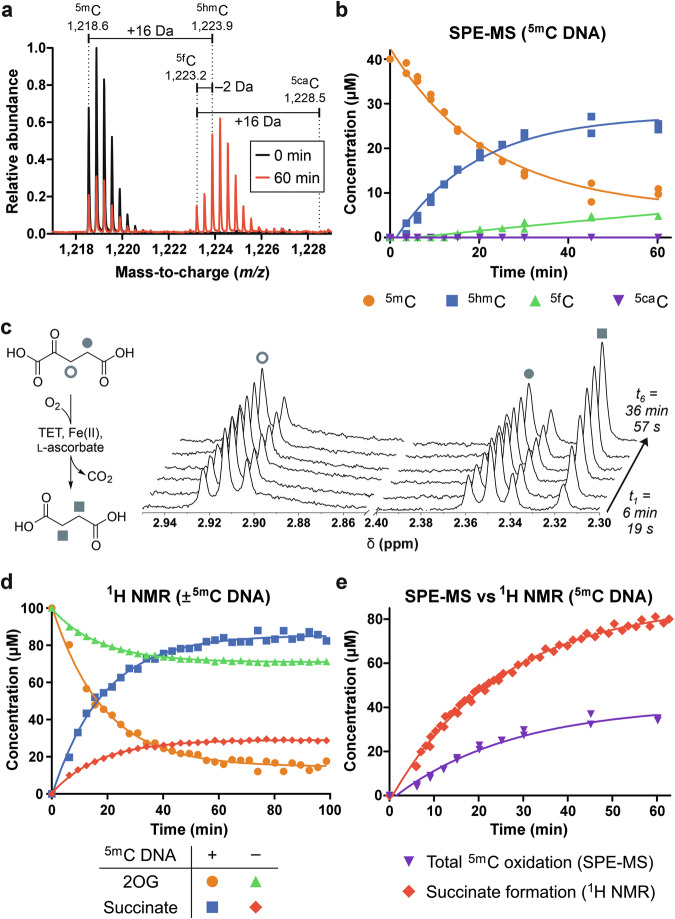
Continuous monitoring of NgTet1 ^5m^C hydroxylase and 2OG decarboxylase activity using SPE-MS and ^1^H NMR assays. **a** Representative spectra for selected SPE-MS assay time points with oligonucleotides observed in the [M − 3H]^3–^ charge state (black, 0 min; red, 60 min). **b** SPE-MS ^5m^C DNA oxidation time course (single DNA strand analyte concentrations reported). **c** Representative spectra for selected ^1^H NMR assay time points (no DNA). **d** ^1^H NMR 2OG decarboxylation time courses in the presence and absence of ^5m^C DNA substrate. **e** Time courses of total ^5m^C oxidation (^5hm^C and ^5f^C production) by SPE-MS and 2OG consumption by ^1^H NMR under DNA substrate-saturating conditions (20 µM ^5m^C DNA). Standard conditions: 5 μM NgTet1, 2 mM ascorbate, 100 μM (NH_4_)_2_Fe(SO_4_)_2_, 100 μM (**d**) or 200 μM (**b,** **e**) 2OG. Data in **b** and **e** plotted from two independent replicates (*n* = 2); ^1^H NMR data in **d** shown as representative curves of two independent replicates.

Human TETs are large (180–230 kDa) multidomain proteins with high structural complexity. Production of full-length recombinant human TETs is challenging and typically requires substantial construct modification (truncation and/or deletion) which may affect substrate oxidation and 2OG turnover dynamics. Thus, a 38 kDa TET homologue from amoeboflagellate *Naegleria gruberi* (NgTet1) was selected as a model enzyme for this work^26^. Full-length NgTet1 was recombinantly expressed and purified from *E. coli* (Figure. S1) and catalytic activity was confirmed using solid-phase extraction-mass spectrometry (SPE-MS)-based assay^33,34^. Successive oxidation of methylated DNA (double-stranded, self-complementary 5*'*-ACC AC^5m^C GGT GGT-3*'* [^5m^C DNA], ^5m^CpG-containing sequence reported as a human TET substrate^24,33^) to ^5hm^C and ^5f^C was observed in the presence of l-ascorbate, Fe(II), and 2OG (Fig. 2a, b). ^5ca^C was not detected after 60 min under the assay conditions tested. ^5m^C DNA substrate *K*_M_^app^ was measured to be 2 µM (Figure. S2).

To investigate the 2OG decarboxylation kinetics of the TETs, a reported ^1^H NMR spectroscopy assay to directly quantify succinate formation for 2OG dioxygenases was adapted^18^. Initially, NgTet1 was incubated with l-ascorbate, Fe(II), and 2OG and analysed by ^1^H NMR spectroscopy, which revealed substantial succinate production over time, indicating uncoupled 2OG turnover in the absence of DNA substrate (Fig. 2c, d). The rate of succinate production was found to be negligible in control experiments when NgTet1 or any of the co-factors were removed (Table S1), indicating that the observed activity was enzymatic, and not due to chemical 2OG decarboxylation pathways, such as ascorbate-mediated succinate formation via a H_2_O_2_ intermediate^35^. Ascorbate was essential for 2OG decarboxylase activity, consistent with reports on other 2OG hydroxylases (Table S1)^36,37^. In the presence of saturating concentrations of substrate ^5m^C DNA (20 µM, 10 × *K*_M_^app^), the rate of 2OG consumption increased, as expected for a combination of substrate-coupled and -uncoupled 2OG turnover (Fig. 2d). However, simultaneous reaction analysis by ^1^H NMR and SPE-MS methods under DNA substrate-saturating conditions revealed that 2OG decarboxylation took place at a substantially faster rate than ^5m^C oxidation (Fig. 2e). Under our assay conditions, after 20 min, ~7.2 nmoles of 2OG had been consumed to oxidise 3.5 nmoles of substrate (a combination of ^5m^C and ^5hm^C DNA substrates), demonstrating poor 2OG decarboxylation coupling even under DNA substrate-saturating conditions.

### 2OG decarboxylation kinetics of NgTet1

**Fig. 3:**
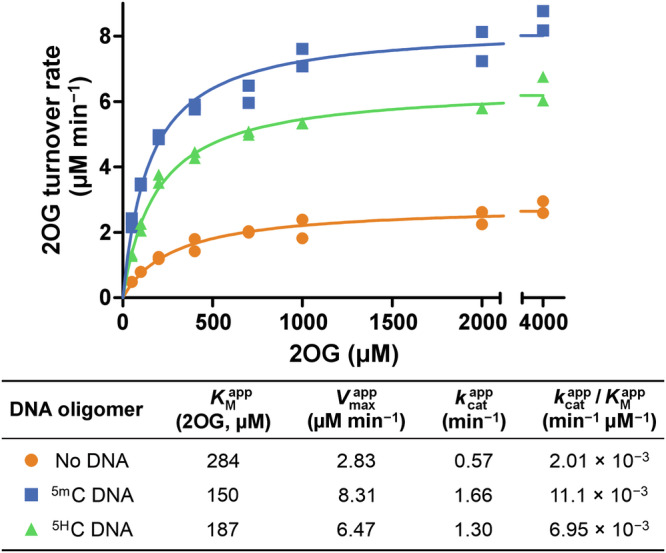
Michaelis-Menten enzyme kinetics plots and corresponding apparent kinetic parameters for 2OG decarboxylation by NgTet1 using ^1^H NMR assay. Standard conditions: 5 μM NgTet1, 2 mM ascorbate, 100 μM (NH_4_)_2_Fe(SO_4_)_2_, 0–4,000 μM 2OG. Double-stranded 12 bp DNA oligomers were added at a final concentration of 20 µM. Data plotted from two independent replicates (*n* = 2). See Figure. S3 for a ^1^H NMR 2OG decarboxylation time course in the presence of ^5H^C DNA.

Next, 2OG kinetic parameters were compared in the presence and absence of substrate-saturating conditions using ^1^H NMR (Fig. 3). Substrate saturation decreased the 2OG *K*_M_^app^ nearly 2-fold (284 to 150 μM), enhanced the maximum 2OG decarboxylation rate 3-fold (*V*_max_^app^ = 2.83 to 8.31 μM min^–‍1^), and increased the apparent catalytic efficiency 5-fold (*k*_cat_^app^/*K*_M_^app^ = 0.00201 to 0.0111 min^−^^1^ μM^−1^). Addition of non-methylated DNA of the same sequence (^5H^C DNA, 5*'*-ACC ACC GGT GGT-3*'*) produced a comparable effect on 2OG turnover to ^5m^C substrate-saturating conditions, but ^5H^C DNA did not undergo enzymatic oxidation (Figure. S4), confirming that it is not a TET substrate. Thus, 2OG decarboxylation kinetics are regulated by both substrate ^5m^C DNA and corresponding non-substrate ^5H^C DNA, suggesting that enzyme–DNA complex formation leads to enhanced 2OG decarboxylation independent of substrate oxidation.

**Fig. 4:**
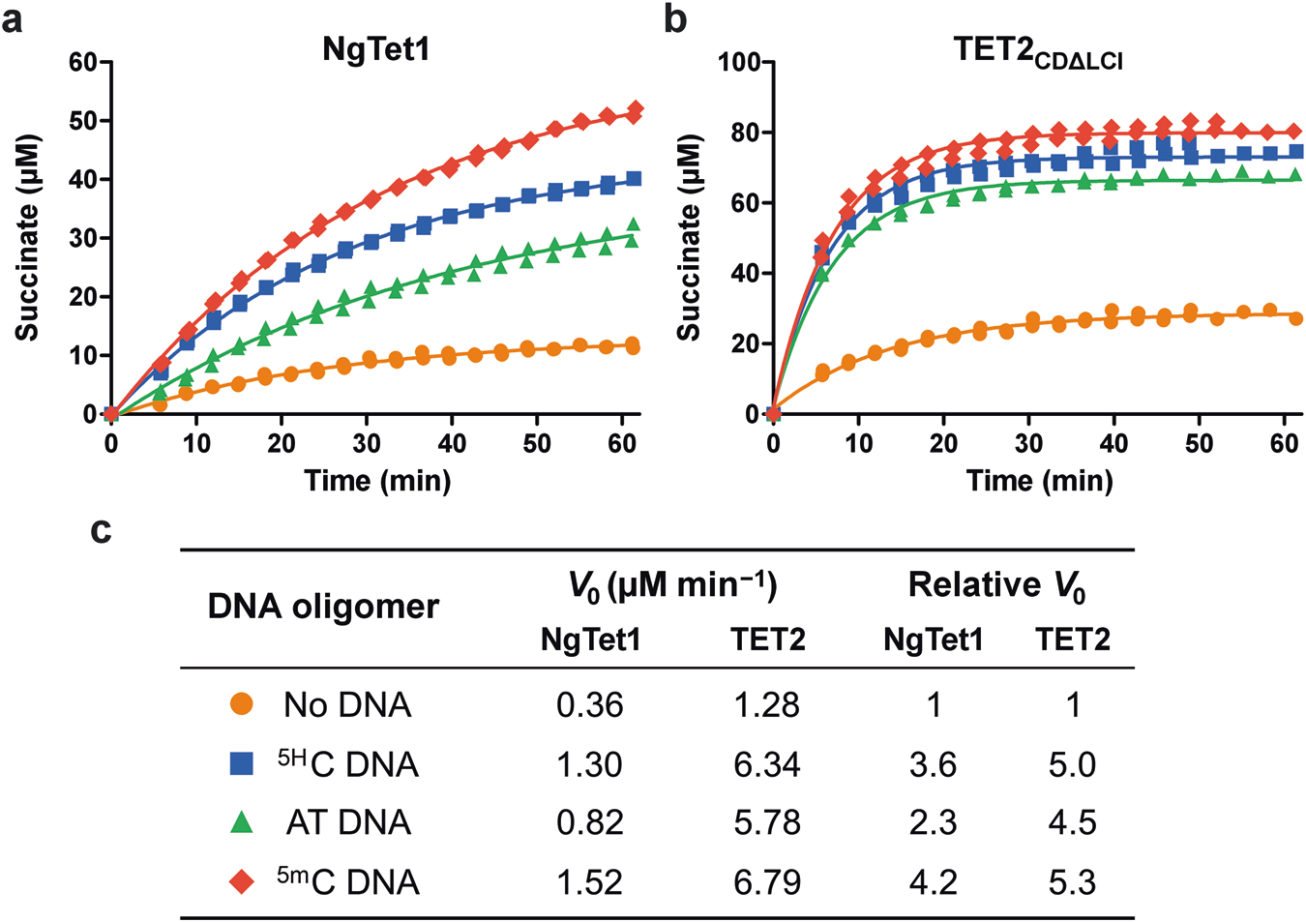
Comparison of TET 2OG decarboxylase activity in the absence of DNA, with ^5H^C, AT, and ^5m^C DNA using ^1^H NMR. **a** NgTet1 activity curves. **b** TET2_CDΔLCI_ activity curves. **c** Tabulated initial reaction rates (*V*_0_) and relative *V*_0_ to reactions without DNA. Standard conditions: 5 μM NgTet1 or TET2_CDΔLCI_, 2 mM ascorbate, 100 μM (NH_4_)_2_Fe(SO_4_)_2_, 100 μM 2OG. Double-stranded DNA oligomers were added at a final concentration of 20 µM. Data plotted from two independent replicates (*n* = 2). Initial rates reported as the mean of two independent replicates (*n* = 2).

To determine if the stimulation of 2OG decarboxylase activity by non-substrate DNA was sequence-specific and contingent on the presence of a CpG site, the central CpG motif in ^5H^C DNA was substituted for ApT (AT DNA, double-stranded self-complementary 5*'*-ACC ACA TGT GGT-3*'*). Addition of AT DNA improved the initial 2OG turnover rate 2.3-fold compared to no DNA conditions, although ^5H^C DNA and ^5m^C DNA were more efficient at accelerating 2OG decarboxylation, producing 3.6- and 4.2-fold enhancements, respectively (Fig. 4a, c). All three DNA oligomers exhibited comparable binding affinities in an AlphaScreen competition assay with a biotinylated ^5m^C-containing dsDNA oligomer (Figure. S5, NgTet1 IC_50_ = 56–60 µM). Reported crystal structures of NgTet1 and TET2_CDΔLCI_ reveal positively charged DNA-interacting patches proximal to their active sites^24,26^, supporting interactions with the DNA phosphate backbone rather than recognition of specific bases as the primary driver of binding. Thus, while the degree of 2OG decarboxylation activation appears to be oligonucleotide sequence-specific, affinity of complexes between TET and DNA oligomers seems to be sequence-agnostic.

### Comparison of NgTet1 and TET2 activity

To investigate the 2OG decarboxylase activity of human TETs, a truncated TET2 catalytic domain construct^24^ (TET2_CDΔLCI_) was recombinantly produced from *E. coli* (Figure. S6), and the same ^5m^C DNA, a reported^24^ substrate of TET2_CDΔLCI_ (*K*_M_ = 1 µM, *k*_cat_^app^ = 0.3 min^−1^)^33^, was used. As with NgTet1, 2OG turnover was observed in the absence of DNA substrate and the rate was enhanced by addition of ^5m^C substrate DNA and non-substrate DNA (^5H^C and AT DNA) (Fig. 4b). However, the rates were less dependent on the oligonucleotide identity for TET2 than in the case of NgTet1, with all three oligonucleotides enhancing initial reaction rates to a similar extent (Fig. 4b, c). While the TET2 binding affinities of all three DNA oligomers were comparable, as in the case of NgTet1 (Figure. S5, TET2_CDΔLCI_ IC_50_ = 4.6–8.9 µM), the observed differences between NgTet1 and TET2 may be partially attributed to discrete DNA sequence preferences and affinities within the TET family^6–8,25^; a direct comparison would require the identification of optimal substrate sequences for each TET protein. Further investigation is required to determine the biological relevance of full-length TET2 2OG decarboxylase activity, as the non-catalytic domains absent from TET2_CDΔLCI_ may affect enzyme specificity and coupling of 2OG turnover^24^.

### Characterisation of TET inhibitors

**Fig. 5:**
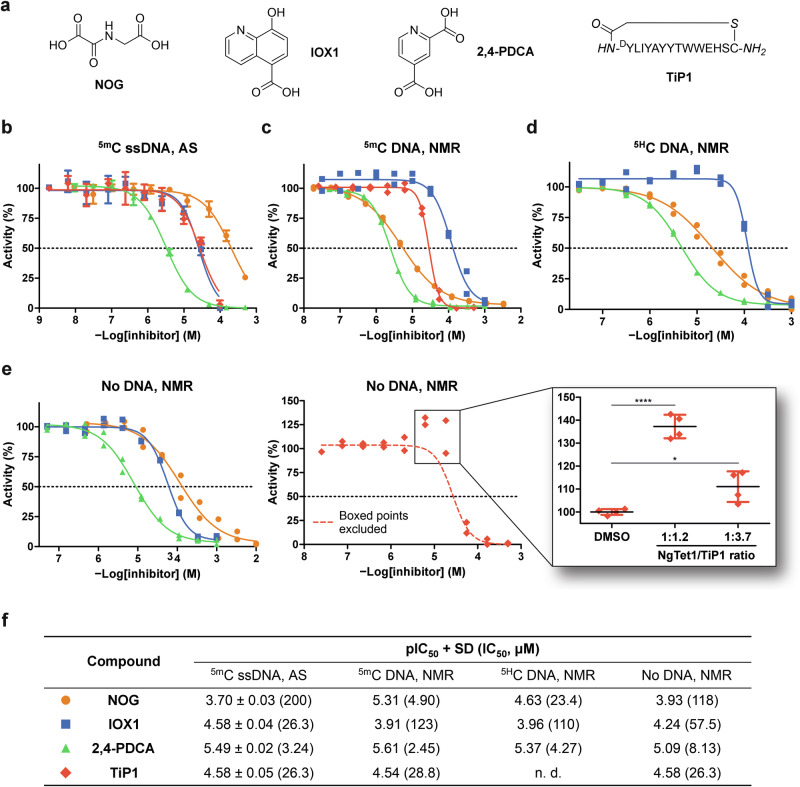
Dual characterisation of inhibitors against NgTet1. **a** Structures of characterised inhibitors (italicised letters in cyclic peptide TiP1 represent individual atoms). Representative dose-response curves for inhibition of NgTet1-catalysed ^5m^C ssDNA oxidation (**b**) using AlphaScreen and 2OG decarboxylation (**c**) with ^5m^C DNA, (**d**) with ^5H^C DNA, and (**e**) with no DNA using ^1^H NMR. **f** Tabulated pIC_50_ and IC_50_ values. Statistical significance of NgTet1 activation (**e** inset, *n* = 4 technical replicates, two-tailed unpaired *t* test): ****p ≤ 0.0001; *p = 0.017. **e** inset data collected separately from TiP1 dose-response curve data. Curve fitting and pIC_50_ determination for TiP1 in **e** were carried out excluding the boxed outliers. Activity was normalised against reactions containing vehicle (DMSO). Standard conditions: 400 nM NgTet1, 10 nM ^5m^C ssDNA, 100 μM ascorbate, 10 μM (NH_4_)_2_Fe(SO_4_)_2_, 10 μM 2OG (AS); 5 μM NgTet1, 0/20 µM ^5m^C or ^5H^C DNA, 2 mM ascorbate, 100 μM (NH_4_)_2_Fe(SO_4_)_2_, 50 μM 2OG (^1^H NMR). AS data shown as the mean of three experimental replicates (*n* = 3; mean ± SD); ^1^H NMR data plotted from two independent replicates (*n* = 2); tabulated data reported as the mean of two and three independent replicates for ^1^H NMR and AS, respectively (*n* = 2–3, SD reported for *n* = 3). N. d.: not determined; AS: AlphaScreen.

Next, the effects of 2OG oxygenase inhibitors on 2OG decarboxylation rates were explored. Known inhibitors of TET ^5m^C hydroxylase activity, including three pan-specific 2OG mimetics (N-oxalylglycine (NOG)^38^, 5-carboxy-8-hydroxyquinoline (IOX1)^39^, and 2,4-pyridinedicarboxylic acid (2,4-PDCA)^40^) and thioether macrocyclic peptide TiP1^32^ (Fig. 5a), were screened for inhibitory activity against NgTet1. Inhibition of ^5m^C hydroxylase activity was evaluated using a reported chemical luminescence and anti-^5hm^C antibody-based AlphaScreen (AS) assay^32^ with ^5m^C ssDNA (single-stranded 32 mer 5’-[Biotin]-TCG GAT GTT GTG GGT CAG ^5m^CGC ATG ATA GTG TA-3*'* reported as an NgTet1 substrate^26^). 2OG mimetics exhibited a range of micromolar potencies (AS IC_50_ = 3–200 µM, Fig. 5b, f), although IOX1 and NOG inhibited NgTet1 to a lesser degree than human paralogues^33^.

In parallel, inhibition of 2OG decarboxylase activity was evaluated using ^1^H NMR assay. Under ^5m^C DNA substrate-saturating conditions (mixed 2OG turnover), the potency of NOG, an isostere of 2OG, improved 40-fold relative to AS assay, while IOX1 potency decreased 5-fold, demonstrating that 2OG competitive inhibitor potencies can be differentially affected by different assays and assay conditions, such as DNA substrate sequence and concentration (Fig. 5c). In the presence of non-substrate ^5H^C DNA (uncoupled 2OG turnover), NOG potency was reduced 5-fold, whereas IOX1 and 2,4-PDCA only showed slight potency changes (<2-fold) (Fig. 5d). In the absence of any DNA (uncoupled 2OG turnover), inhibitory potencies were comparable to ^5m^C ssDNA-based AS assay results (NMR IC_50_ = 8–‍118 µM, Fig. 5e). While the potency of small molecule 2OG mimetics varied substantially depending on the assay, TiP1 potency was consistent across all assays (IC_50_ = 26–29 µM). An increase in uncoupled 2OG decarboxylation activity was observed when approaching 1:1 stoichiometry between NgTet1 and cyclic peptide TiP1 in the absence of DNA oligomers. At 1:1.2 and 1:3.7 ratios of NgTet1 to TiP1 (5 µM NgTet1), TiP1 stimulated 2OG turnover in a statistically significant manner (Fig. 5e inset), although further titration of TiP1 resulted in activity inhibition. MALDI-TOF MS analysis of the reaction after quenching revealed no changes to TiP1 (Figure. S7), demonstrating that the catalytic activity enhancement was not due to the recognition of TiP1 as substrate. At 1:1.2 stoichiometry of NgTet1/TiP1, TiP1 increased the maximal 2OG turnover velocity 2-fold while reducing 2OG affinity proportionally (Figure. S8); for comparison, ^5H^C DNA increased both *V*_max_ and 2OG affinity (Fig. 3). Accordingly, ^5H^C DNA was a more potent stimulator of uncoupled 2OG decarboxylase activity than TiP1, with 188% and 137% activity observed at 1.2 equivalents of ^5H^C DNA and TiP1, respectively (Figure. S9). TiP1 did not enhance 2OG decarboxylase activity in the presence of DNA substrate, potentially indicating mutual binding exclusivity of TiP1 and DNA oligomers. Similarly, in the absence of substrate, substrate competitors elevated 2OG turnover rates in prolyl hydroxylases^19,41^, suggesting an analogous substrate-competitive mechanism of action for TiP1.

## Discussion

Overall, analysis of TET DNA oxidation and 2OG decarboxylase activities using ^1^H NMR and SPE-MS assays provided insight into the interplay between substrate coupled and uncoupled catalytic activity. Our work highlights the unusually poor control of 2OG decarboxylation coupling to substrate oxidation in TETs, in line with the trends observed in uncoupled decarboxylation across 2OG oxygenases using a bioluminescent succinate detection assay^23^. While the biological relevance of the high levels of uncoupled 2OG turnover in TETs is unclear, it suggests potential secondary roles for TETs beyond DNA oxidation/demethylation, such as modulation of TCA cycle intermediate levels (i.e., 2OG and succinate). This could have wide-ranging implications, including modulation of local intracellular 2OG dioxygenase activity, which can be inhibited by high levels of succinate^42–44^. Notably, the uncoupled 2OG consumption rates of TETs were further enhanced upon binding to substrate and non-substrate DNA, in both CpG and non-CpG contexts. While NgTet1 was used in this study as a model system for detailed TET kinetics, human TET2 showed similar trends, demonstrating that 2OG decarboxylase activity is strongly affected by oligonucleotide binding.

Development of a ^1^H NMR assay also enabled the study of the effect of TET inhibitors on coupled and uncoupled 2OG turnover rates. Inhibitor potencies were largely dependent on assay conditions in the case of 2OG mimetics NOG, IOX1, and 2,4-PDCA, but not macrocyclic peptide TiP1, indicating that 2OG competitive small molecules can differentially inhibit the rates of substrate oxidation and coupled/uncoupled 2OG turnover by TETs. Notably, TiP1 enhanced 2OG decarboxylase activity in the absence of DNA oligomers, mimicking the effects of oligonucleotide binding. 2OG competitive inhibitors or substrate competitive TET ligands, such as oligonucleotides or inhibitors, can thus influence the uncoupled 2OG decarboxylation rates, which may result in previously underappreciated changes to the balance of intracellular 2OG/succinate levels. Given the central role of TETs in epigenetic regulation and the observed TET dysregulation in multiple diseases, our results suggest that further investigation into the cellular effects of TET activity beyond ^5m^C oxidation and the resulting epigenetic consequences would be of particular interest.

## Methods

### Recombinant NgTet1 production

A pET-28 vector containing a gene coding for N–‍terminally His_6_-tagged full-length *Naegleria gruberi* NgTet1 (321 a. a.) protein with a thrombin cleavage site was expressed in *E. coli* as reported with slight modifications^26^. Cultures were grown at 37 °C in 2× TY medium (1.6% *w/v* tryptone, 1% *w/v* yeast extract, 0.5% *w/v* NaCl) to OD_600_ 0.8, when the temperature was reduced to 16 °C and isopropyl β-d-1-thiogalactopyranoside (IPTG, 0.5 mM) was added to induce expression (18 h). Cells were harvested (7741 × *g*, 4 °C, 20 min) and stored at −80 °C. The cells were thawed and re-suspended in 4 volumes of 20 mM HEPES (pH 7.5), 500 mM NaCl, 20 mM imidazole, 0.5 mM tris(2-carboxyethyl)phosphine (TCEP), 0.1% *v/v* Benzonase^®^, and 1× Calbiochem^®^ protease inhibitor cocktail set III (EDTA-free) and lysed using sonication. The lysate was clarified by centrifugation (63,988 × *g*, 4 °C, 30 min). His_6_-tagged NgTet1 was captured from the lysate supernatant using Ni affinity chromatography (HisTrap HP) and further purified by size exclusion chromatography (Superdex^®^ 75) in 20 mM HEPES (pH 7.5), 150 mM NaCl, 0.5 mM TCEP. The purified protein was stored at a concentration of 18 mg mL^−1^ in gel filtration buffer.

### Recombinant TET2_CDΔLCI_ production

A modified pET-28b vector containing a gene coding for the catalytic domain of TET2 (TET2_CD_) with an insertion-deletion at the low complexity insert region (LCI, residues 1129–1936, with residues 1481–1843 replaced with linker GGGGSGGGGSGGGGS) fused to an N–terminal His_6_-FLAG-[thrombin cleavage site]-SUMO tag was expressed in *E. coli* based on the reported procedure^24^. Cultures were grown at 37 °C in TB medium (2% *w/v* tryptone, 2.4% *w/v* yeast extract, 0.4% *v/v* glycerol, 24.6 mM KH_2_PO_4_, 75.4 mM K_2_HPO_4_ [pH 7.4]) to OD_600_ 1.0, when the temperature was reduced to 16 °C and IPTG (0.5 mM) was added to induce expression (18 h). Cells were harvested (7741 × g, 4 °C, 20 min) and stored at −80 °C. The cells were thawed and re-suspended in 5 volumes of 20 mM Tris (pH 8.0), 500 mM NaCl, 10 mM imidazole, 0.5 mM TCEP, 0.1% *v/v* Benzonase^®^, and 1× Calbiochem^®^ protease inhibitor cocktail set III (EDTA-free) and lysed using high-pressure homogenisation. The lysate was clarified by centrifugation (63,988 × *g*, 4 °C, 30 min). His_6_-tagged TET2_CDΔLCI_ fusion was captured from the lysate supernatant using Ni affinity chromatography (HisTrap HP). The captured protein was dialysed into low-imidazole buffer (20 mM Tris (pH 8.0), 500 mM NaCl, 10 mM imidazole), and the fusion tag was removed by treatment with Ulp1 protease at 4 °C for 16 h. The His_6_-containing solubility tag was re-captured on a Ni affinity column and the cleaved TET2_CDΔLCI_ was further purified by heparin affinity chromatography (Heparin HP), loading in 50 mM Tris (pH 8.0), 50 mM NaCl, 0.5 mM TCEP and gradient eluting with 50 mM Tris (pH 8.0), 2 M NaCl, 0.5 mM TCEP (0–100%). TET2_CDΔLCI_ was further purified by size exclusion chromatography (Superdex^®^ 200) in 20 mM Tris (pH 8.0), 300 mM NaCl, 0.5 mM TCEP. The purified protein was stored at a concentration of 20 mg mL^−1^ in gel filtration buffer.

### SPPS synthesis of TiP1

TiP1 (Figure. S11) was synthesised on a 100 μmole scale on a Liberty Blue automated peptide synthesiser (CEM) using standard fluorenylmethyloxycarbonyl (Fmoc) solid-phase peptide synthesis (SPPS) as previously described^32^. Briefly, linear TiP1 was synthesised on a Rink amide 4-methylbenzhydrylamine (MBHA) resin. The α-N–amine was chloroacetylated using chloroacetic anhydride (10 eq.) in DMF at room temperature for 3 h, the resin was washed (DMF and CH_2_Cl_2_), and air-dried. The peptide was cleaved from the resin by treatment with a 37:1:1:1 mixture of trifluoroacetic acid (TFA)/1,3-dimethoxybenzene/triisopropyl silane (TIPS)/H_2_O at room temperature for 3 h. The peptide was precipitated from ice-cold diethyl ether and pelleted by centrifugation (4000 × *g*, 10 min, 4 °C).The pellet was further washed with ice-cold diethyl ether (4×), air-dried, and the crude was lyophilised from a solution of 1:1 H_2_O/MeCN. The resulting pellet was dissolved in DMSO (20 mg mL^−1^), and the solution was basified by the addition of *N*,*N*–diisopropylethylamine (DIPEA) to facilitate intramolecular thioether formation (40 °C, 1.5 h). TiP1 was purified to >99.0% purity by reverse-phase HPLC (Phenomenex Gemini^®^ 5 μm NX-C18 110 Å LC column, H_2_O/MeCN solvent system supplemented with 0.1% *v/v* TFA). Peptide purity and identity was verified by LC–UV and HR–MS (Figure. S12) using an ACQUITY Ultra Performance LC system coupled to a Waters Xevo G2-XS Q-TOF mass spectrometer equipped with an ESI LockSpray™ source and an ACQUITY UPLC HSS T3 Column (100 Å, 1.8 μm, 2.1 × 100 mm).

### ^1^H NMR spectroscopy

All ^1^H NMR spectra were recorded at 25 °C using D_2_O as a reference for internal deuterium lock on a Bruker Avance III 700 MHz equipped with an inverse TCI cryoprobe in 3 or 5 mm diameter tubes with a total sample volume of 160 or 565 μL, respectively. The water signal was reduced using Perfect Echo excitation sculpting suppression. Chemical shifts are reported in ppm relative to D_2_O (δ_H_ = 4.70 ppm).

### Continuous reaction monitoring (^1^H NMR)

Spectrometer settings were determined using a control sample containing all reaction components excluding enzyme. TET enzyme was added to an identical sample, and the resulting assay mixture was transferred to an NMR tube before deposition into the instrument. The final reaction mixtures contained 5 μM NgTet1/TET2_CDΔLCI_, 2 mM sodium l-ascorbate, 100 μM (NH_4_)_2_Fe(SO_4_)_2_, and 50–4,000 μM disodium 2OG in 50 mM Tris-d_11_ (pH 7.5), 10% *v/v* D_2_O; ^5m^C, ^5H^C, or AT DNA were added to a final concentration of 20 μM of double-stranded DNA (total ^5m^C concentration of 40 μM) as necessary. Data acquisition was started following a brief spectrometer setting optimisation (up to 200 s delay between enzyme addition and data acquisition); 8–32 scans were accumulated for a single spectrum, corresponding to 65–‍179 s of acquisition time. Spectrum recordings were carried out consecutively without delay. Data were processed using automated routines on MestReNova v14.2.0; signals of interest were integrated with absolute intensity scaling. ^1^H NMR time courses reported in figures are representative results from two or three independent replicates.

### End-point enzyme inhibition assays (^1^H NMR)

NgTet1 was pre-incubated with inhibitor dilution series in DMSO-d_6_ at room temperature for 10 min (50 mM Tris-d_11_ [pH 7.5], 2% *v/v* DMSO-d_6_). Reactions were initiated by the addition of appropriate cofactors and DNA. The final reaction mixtures contained 5 μM NgTet1, 2 mM sodium l-ascorbate, 100 μM (NH_4_)_2_Fe(SO_4_)_2_, 50 μM disodium 2OG, and 1% *v/v* DMSO-d_6_ in 50 mM Tris-d_11_ [pH 7.5], 10% *v/v* D_2_O; ^5m^C or ^5H^C DNA was added to a final concentration of 20 μM of double-stranded DNA (40 μM of ^5m^C) as necessary. The reactions were quenched by the addition of 9 mM NOG and 9 mM ZnCl_2_ in 90 mM Tris-d_11_ [pH 7.5] (20 μL) within the enzyme linear activity window (determined in Figure. S10). Samples were transferred to NMR tubes and subjected to ^1^H NMR analysis. Data were processed as described above. Inhibition data was normalised to reactions containing vehicle only (1% *v/v* DMSO-d_6_) and fitted using “log(inhibitor) vs. response - Variable slope (four parameters)” non-linear regression model on GraphPad Prism 5.

### AlphaScreen ^5hm^C detection assay

AlphaScreen™ IgG (Protein A) Detection Kit was used to evaluate inhibitor potency by quantifying ^5hm^C levels in reaction mixtures following reported procedures^33,34^ using an anti-^5hm^C antibody (Active Motif, RRID: AB_10013602). Compounds in DMSO (100 nL) were pre-dispensed in a logarithmically linear concentration gradient onto 384-well ProxiPlates using an Echo® 550 acoustic liquid handler (Labcyte). NgTet1 was pre-incubated with the inhibitor dilution series in DMSO at room temperature for 10 min. Reactions were initiated by the addition of appropriate cofactors and DNA. The final reaction mixtures (10 μL) contained 400 nM NgTet1, 10 nM ^5m^C ssDNA, 100 μM sodium l-ascorbate, 10 μM (NH_4_)_2_Fe(SO_4_)_2_, 10 μM disodium 2OG, and 1% *v/v* DMSO in assay buffer (50 mM HEPES [pH 7.3], 150 mM NaCl, 0.1% *v/v* BSA, 0.01% *v/v* Tween^®^ 20). The reactions were quenched by the addition of 30 mM EDTA (pH 4.2), 358 mM NaCl (5 μL) within the enzyme linear activity window (determined in Fig. S10). Concurrently, a mixture containing the anti-^5hm^C antibody (1:2000 dilution) and AlphaScreen beads (1:62.5 dilution) in assay buffer was prepared and incubated at room temperature for 30 min. The AlphaScreen bead/antibody mixture was added to the quenched NgTet1 reactions (5 μL), and the complete mixture was incubated at room temperature for 1 h. Chemiluminescence was measured on a PHERAstar FS/FSX (BMG Labtech) equipped with an AlphaScreen 680 570 module. All AlphaScreen bead manipulations were performed under subdued lighting. Inhibition data was normalised to reactions containing vehicle only (1% *v/v* DMSO) and fitted using “log(inhibitor) vs. response—Variable slope (four parameters)” non-linear regression model on GraphPad Prism 5.

### AlphaScreen competition assay

AlphaScreen™ Histidine (Nickel Chelate) Detection Kit was used to evaluate displacement of non-biotinylated competitor DNA oligomers by a biotinylated double-stranded 32 bp DNA oligomer (^5m^C dsDNA)^33,34^. Competitor DNA oligomers in assay buffer were manually dispensed onto 384-well ProxiPlates (5 μL) in a 2-fold dilution series. His_6_-tagged NgTet1 or TET2_CDΔLCI_ in assay buffer was added (5 μL), and the mixture was pre-incubated with the competitor DNA at room temperature for 10 min. Cofactors and biotinylated ^5m^C dsDNA in assay buffer were added (5 μL), and the mixture was further incubated at room temperature for 10 min. A mixture of AlphaScreen beads (1:62.5 dilution) in assay buffer was added (5 μL), and the full mixture was incubated at room temperature for 1 h. The final mixtures (20 μL) contained 100 nM NgTet1 (50 nM ^5m^C dsDNA) or 12.5 nM TET2_CDΔLCI_ (12.5 nM ^5m^C dsDNA), 50 μM sodium l-ascorbate, 5 μM (NH_4_)_2_Fe(SO_4_)_2_, and 5 μM disodium NOG in assay buffer (50 mM HEPES [pH 7.3], 150 mM NaCl, 0.1% *v/v* BSA, 0.01% *v/v* Tween^®^ 20). For the assay interference counter screen, His_6_-tagged TET protein and biotinylated ^5m^C dsDNA were replaced with 1.6 nM biotinylated His_6_ peptide. Chemiluminescence was measured on a PHERAstar FS/FSX (BMG Labtech) equipped with an AlphaScreen 680 570 module. All AlphaScreen bead manipulations were performed under subdued lighting. Inhibition data was normalised to reactions containing no competitor and fitted using “log(inhibitor) vs. response - Variable slope (four parameters)” non-linear regression model on GraphPad Prism 5.

### SPE-MS

SPE-MS spectra were recorded on an Agilent RapidFire 365 High-throughput Mass Spectrometry system coupled to an Agilent 6550 iFunnel Q-TOF mass spectrometer following reported procedures^33,34^. A RapidFire C_4_ (type A) solid-phase extraction (SPE) cartridge (Agilent, Cat#: G9203A) was used for analyte separation. A binary solvent system was used. Solvent A: 6 mM octylammonium acetate (prepared as described previously)^45^ in H_2_O; solvent B: 80% *v/v* MeCN in H_2_O. Samples were aspirated into the sample loop using vacuum (40 μL) from a 96/384-well plate and loaded directly onto the SPE cartridge. The cartridge was washed with solvent A (4,000 ms, 1.5 mL min^−1^), and the analyte was eluted with solvent B (4000 ms; 1.25 mL min^−1^) onto the Q-TOF MS. Following each sample injection, the SPE cartridge was flushed with 3–6 cycles of H_2_O and MeCN to remove residual analyte before re-equilibration into solvent A. Q-TOF settings for DNA oligomer analysis were as follows: drying gas temperature: 280 °C; drying gas flow: 13 L min^−1^; nebuliser gas pressure: 40 psig; sheath gas temperature: 350 °C; sheath gas flow: 12 L min^−1^; capillary voltage (Vcap): 4000 V; nozzle voltage: 0 V; fragmentor voltage: 250 V; spectrum acquisition rate: 5 Hz. Data was processed using Agilent MassHunter Qualitative Analysis™ (B.07.00) and RapidFire Integrator software.

### Continuous reaction monitoring (SPE-MS)

A sample containing all reaction components excluding NgTet1 was prepared in a 96-deep well plate. The reaction was initiated at room temperature by the addition of NgTet1 and the resulting mixture was sampled at pre-determined intervals (typically 180 s). The final reaction mixtures contained 500 nM NgTet1, 0.5–10 μM ^5m^C DNA, 200 µM sodium l-ascorbate, 20 μM (NH_4_)_2_Fe(SO_4_)_2_, and 500 µM disodium 2OG in 50 mM Tris (pH 7.1).

### End-point analysis for tandem activity detection (SPE-MS)

Small portions of reaction mixtures prepared for ^1^H NMR continuous reaction monitoring experiments (5 μL) were quenched by the addition of 2,4-PDCA (1.1 mM in 50 mM Tris [pH 7.5], 45 μL). The final quenched mixtures contained 500 nM NgTet1, 2 μM ^5m^C DNA, 200 µM sodium l-ascorbate, 10 μM (NH_4_)_2_Fe(SO_4_)_2_, 20 µM disodium 2OG, and 1 mM 2,4-PDCA in 45 mM Tris, 5 mM Tris-d_11_ (pH 7.5), 1% *v/v* D_2_O. The samples were analysed by SPE-MS.

### Reporting summary

Further information on research design is available in the Nature Portfolio Reporting Summary linked to this article.

## Supplementary information

Supporting information

Description of Additional Supplementary Files

Supplementary Data 1

Reporting summary

## Data Availability

The authors declare that the data supporting the findings of this study are available within the paper and its supplementary information files. Further processed and unprocessed data, including MALDI-TOF files, SPE-MS files, AS luminescence files, and SDS-PAGE gel images, is available upon request.
