# Supplementary material for: Unravelling 2-oxoglutarate turnover and substrate oxidation dynamics in 5-methylcytosine-oxidising TET enzymes
Source: Commun Chem. 2024 Dec 20;7:305. doi: 10.1038/s42004-024-01382-1 (PMC11662004; doi:10.1038/s42004-024-01382-1)
Supplement: Supplementary file 1 — Supporting information [file 42004_2024_1382_MOESM1_ESM.pdf]

Supplementary Information

Unravelling 2-Oxoglutarate Turnover and Substrate Oxidation Dynamics in 5-Methylcytosine-Oxidising TET Enzymes

Klemensas Šimelis[1], Roman Belle[2], and Akane Kawamura[1,2]*

[1] Chemistry Research Laboratory, Department of Chemistry, University of Oxford, 12 Mansfield Road, Oxford, OX1 3TA, United Kingdom

[2] Chemistry - School of Natural and Environmental Sciences, Bedson Building, Newcastle University, Newcastle upon Tyne, NE1 7RU, United Kingdom

Corresponding author email: akane.kawamura@newcastle.ac.uk

Supplementary Methods

General materials

All chemicals were purchased from Sigma Aldrich unless otherwise stated. Reagents obtained from other sources are as follows: anti-^{5hm}C antibody (pAb, rabbit, Active Motif, Cat#: 39769, RRID: AB_10013602), AlphaScreen® IgG Detection Kit (Protein A) (Perkin Elmer, Cat#: 6760617M), AlphaScreen Histidine (Nickel Chelate) Detection Kit (Perkin Elmer, Cat#: 6760619M), ProxiPlate-384 microplates (light gray, shallow-well 384-well, Perkin Elmer, Cat#: 6008350), bovine serum albumin (BSA, Stabilizer, 7.5%, DTPA-purified, Perkin Elmer, Cat#: CR84-100), Tween® 20 (Promega, Cat#: H5151), dimethyl sulfoxide (DMSO, Fisher Chemical, Cat#: D/4120/PB08), IOX1 (Active Motif, Cat#: 14057), *N*–oxalylglycine (NOG, Cayman Chemical, Cat#: 13944), 2,4-pyridinedicarboxylic acid (2,4-PDCA, Sigma Aldrich, Cat#: 04473). LC-MS grade water and acetonitrile used for SPE-MS were purchased from Supelco (LiChroSolv®, Cat#: 1.15333.2500 and 1.00030.2500, respectively).

DNA oligomer information

Name	Assay	Hybridisation	Length	Modification	Sequence (5'–3')	Source	Purity
^{5H}C DNA	^{1}H NMR	Double-stranded self-complementary	12 bp	None	ACC ACC GGT GGT	Invitrogen	>85% (HPLC)
AT DNA	^{1}H NMR	Double-stranded self-complementary	12 bp	None	ACC ACA TGT GGT	Invitrogen	>85% (HPLC)
^{5m}C DNA	^{1}H NMR, SPE-MS	Double-stranded self-complementary	12 bp	5-Methylcytosine	ACC AC**^{5m}C** GGT GGT	ATDBio	>98% (HPLC)
^{5m}C ssDNA	AS	Single-stranded	32 nt	Biotinylation, 5-methylcytosine	[Biotin]-TCG GAT GTT GTG GGT CAG **^{5m}C**GC ATG ATA GTG TA	Prepared in-house	>98% (HPLC)
^{5m}C dsDNA	AS	Double-stranded	32 bp	Biotinylation, 5-methylcytosine	Forward strand: same as ^{5m}C ssDNA Reverse strand: TA CAC TAT CAT GCG CTG ACC CAC AAC ATC CGA	Prepared in-house	>98% (HPLC)

Supplementary Figures

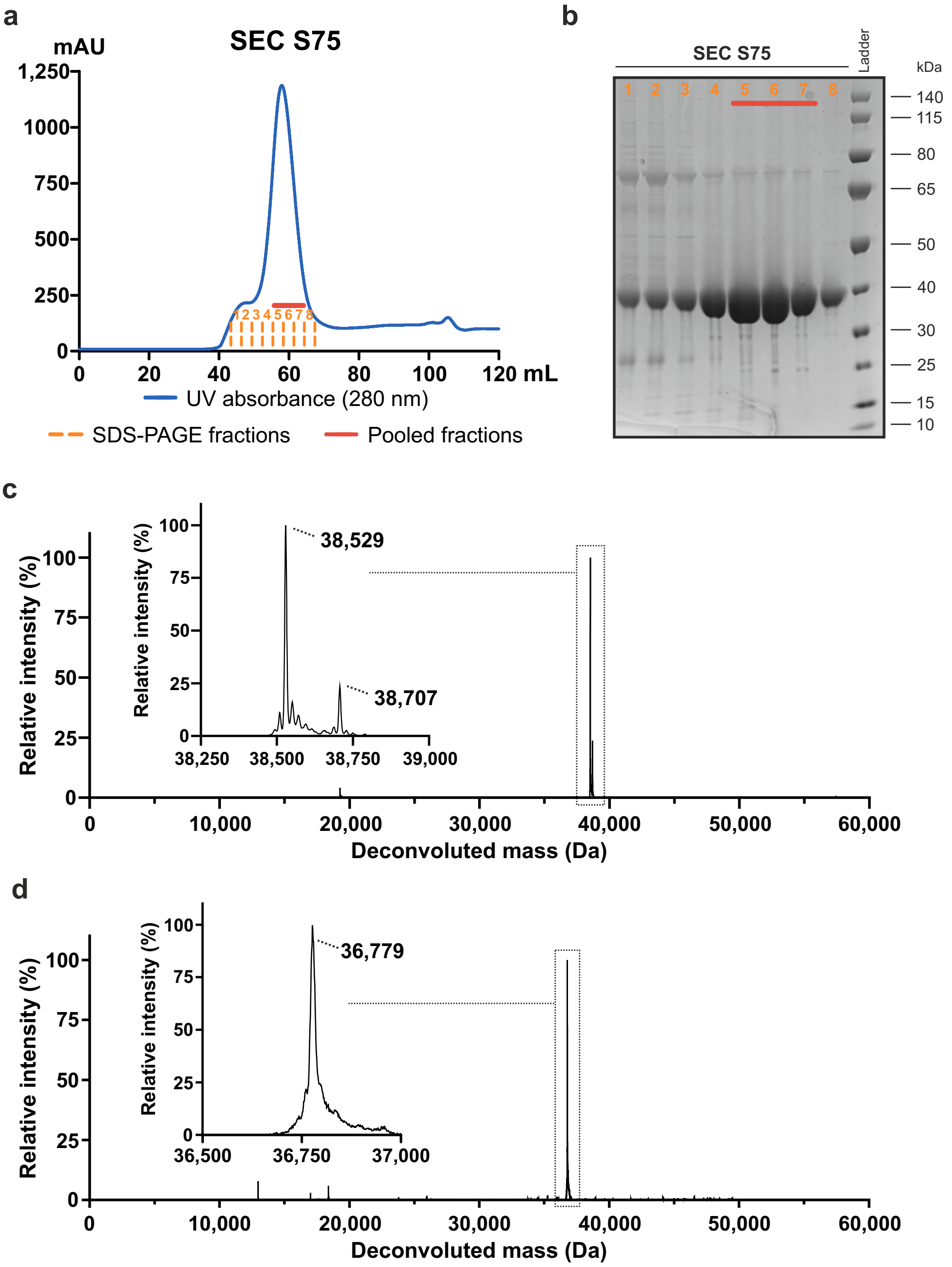

Fig. S1 Recombinant production of NgTet1 in *E. coli* BL21(DE3) strain. **a** 280 nm elution profile of Superdex® 75 size exclusion chromatography step (SEC S75). **b** SDS-PAGE analysis of SEC S75 fractions; red bar indicates pooled fractions. **c** LC-MS analysis of purified NgTet1 (theoretical mass for N–terminally His_6-tagged NgTet1: 38,531 Da, observed mass: 38,529 Da; theoretical mass for α-N–gluconoylated NgTet1: 38,708 Da; observed mass: 38,707 Da). **d** LC-MS analysis of His_6 tag-free NgTet1 following thrombin digestion (theoretical mass: 36,780 Da, observed mass: 36,779 Da). A +178 Da species was observed in the mass spectrum of purified His_6-tagged NgTet1 (**c**, 38,707 Da), attributed to the cleavage of initiator methionine[1] followed by spontaneous α-N–gluconoylation[2] of the N–terminal Gly preceding the His_6 tag (H_2N–$GSSH_6$…). Treatment of His_6-tagged NgTet1 with thrombin protease to remove the affinity tag resulted in the convergence of the two peaks (**d**), confirming the modification is situated at the N–terminus.

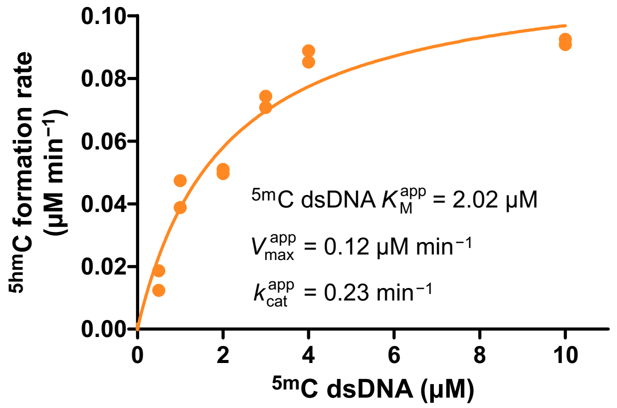

Fig. S2 Michaelis-Menten enzyme kinetics plot and corresponding parameters for ^{5m}C DNA substrate oxidation by NgTet1 using SPE-MS assay. K_M, V_{max}, and k_{cat} values are reported as apparent due to the use of non-saturating 2OG cofactor concentration. Conditions: 500 nM NgTet1, 0.5–10 µM ^{5m}C DNA (double-stranded self-complementary 5'-ACC AC^{5m}C GGT GGT-3', one ^{5m}C unit per strand), 200 µM L-ascorbate, 20 µM $(NH_4)_2Fe(SO_4)_2$, 500 µM 2OG, 50 mM Tris, pH 7.1. Data plotted from two independent replicates (n = 2).

Table S1 2OG decarboxylation observed under various experimental conditions as determined using ^{1}H NMR assay.

Type	L-Ascorbate	Fe(II)	NgTet1	µM succinate (% turnover)	
				After 1 h	After 8 h
Non-enzymatic	-	-	-	0.3 (0.1%)	0.4 (0.1%)
	+	-	-	6.1 (1.2%)	14.6 (2.9%)
	-	+	-	2.6 (0.5%)	3.0 (0.6%)
	+	+	-	2.0 (0.4%)	3.3 (0.7%)
Enzymatic	-	-	+	0 (0%)	n.d.
	+	-	+	3.7 (0.8%)	n.d.
	-	+	+	7.3 (1.5%)	n.d.
	+	+	+	35.4 (8.8%)*	n.d.

2OG was present in all mixtures (500 µM). Non-enzymatic 2OG decarboxylation was marginal in the presence of a combination of L-ascorbate and Fe(II). NgTet1-catalysed uncoupled 2OG decarboxylation exhibited absolute dependence on both L-ascorbate and Fe(II) for sustained activity. Conditions: 5 µM NgTet1, 2 mM L-ascorbate, 100 µM Fe(II), 500 µM 2OG, 50 mM Tris-d_{11}, pH 7.5, 10% *v/v* D_2O. N.d., not determined. Fe(II), $(NH_4)_2Fe(SO_4)_2$. Data from single replicates reported.
* Data was collected in the presence of 400 µM 2OG after 25 min.

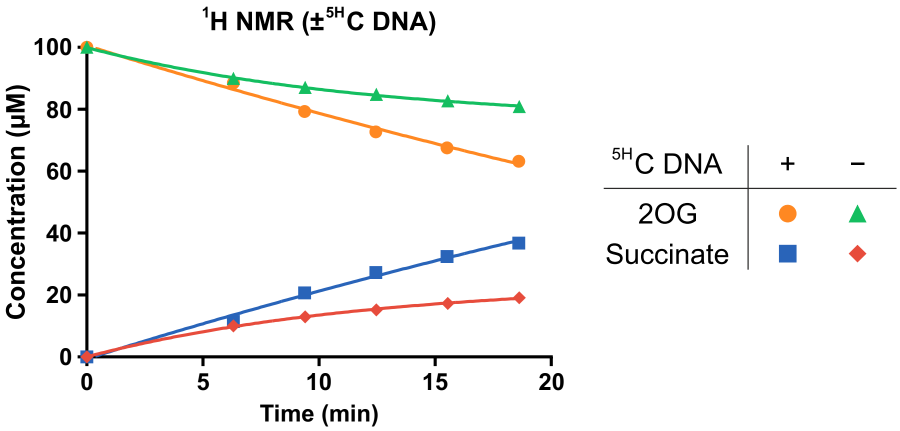

Fig. S3 ^{1}H NMR 2OG decarboxylation time course in the presence and absence of non-substrate ^{5H}C DNA. Conditions: 5 μM NgTet1, 2 mM ascorbate, 100 μM $(NH_4)_2Fe(SO_4)_2$, 100 μM 2OG, 0/20 μM ^{5H}C DNA, 50 mM Tris, pH 7.1. Data shown as representative curves of two independent replicates.

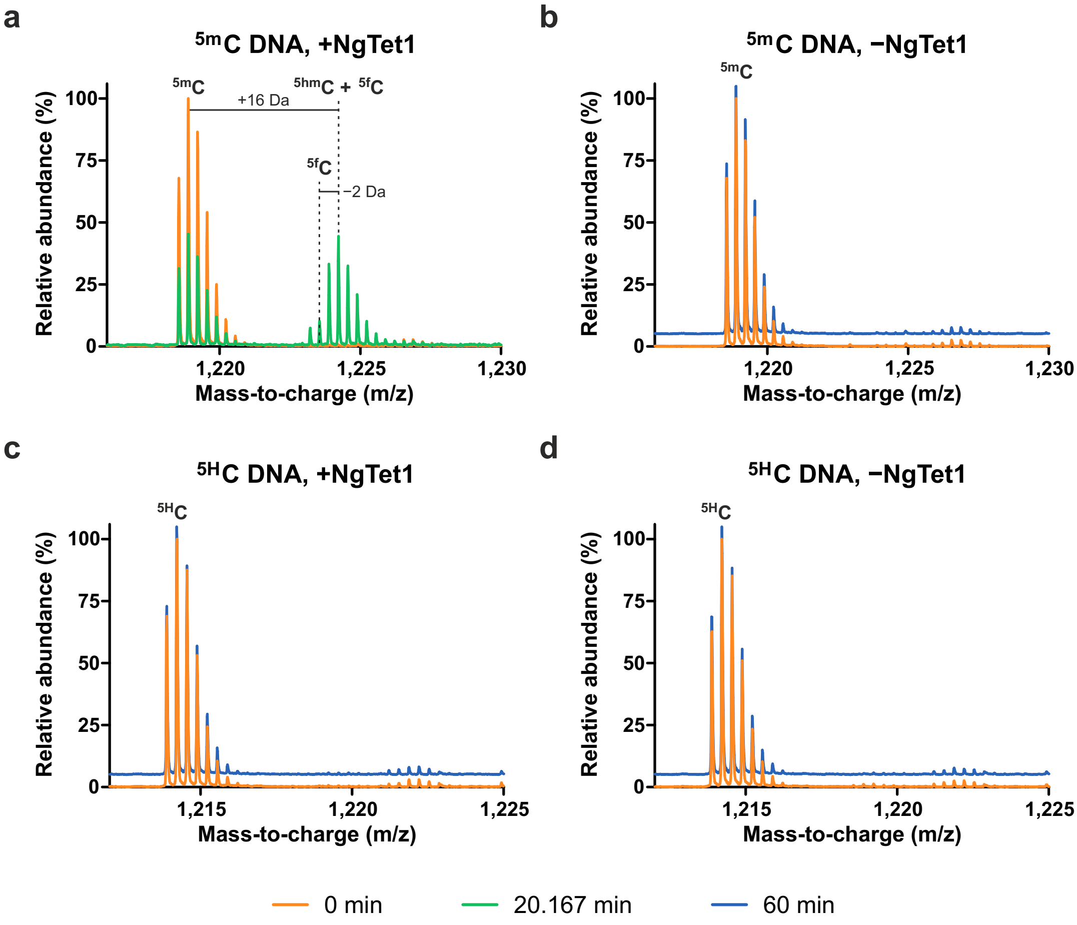

Fig. S4 SPE-MS analysis of ^{5m}C and ^{5H}C DNA in the presence or absence of NgTet1 under assay conditions. Spectra in **b**, **c**, and **d** are vertically staggered by 5 units to avoid peak obfuscation. Peaks represent $[M–3H]^{3-}$ species. Conditions: 5 μM NgTet1, 20 μM ^{5m}C/^{5H}C DNA, 2 mM L-ascorbate, 100 μM $(NH_4)_2Fe(SO_4)_2$, 200 μM 2OG, 50 mM Tris-d_{11}, pH 7.5, 10% *v/v* D_2O.

a

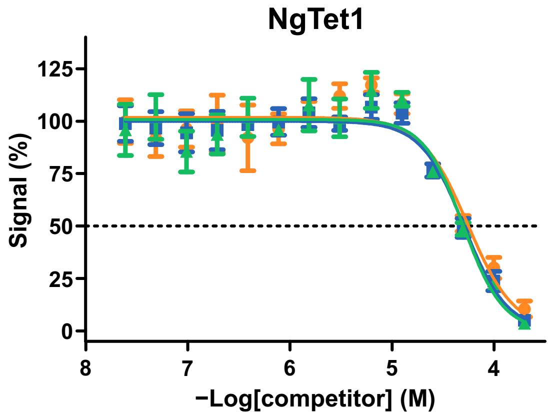

b

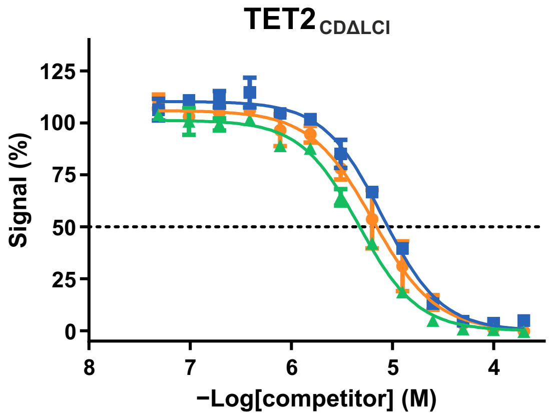

c

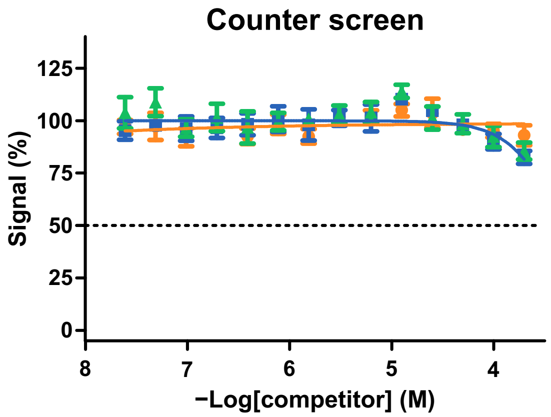

d

Competitor	pIC_{50} (IC_{50}, µM)	
	NgTet1	$TET2_{CD\Delta LCI}$
● **^{5m}C DNA**	4.22 (60.3)	5.13 (7.41)
■ **^{5H}C DNA**	4.24 (57.5)	5.05 (8.91)
▲ **AT DNA**	4.25 (56.2)	5.34 (4.57)

Fig. S5 TETs bind to DNA in a sequence- and modification-independent manner. A binding assay between a biotinylated ^{5m}C dsDNA oligomer and His_6-tagged TET was developed using AlphaScreen Histidine (Nickel Chelate) assay. Double stranded self-complementary non-biotinylated 12 bp ^{5m}C, ^{5H}C, and AT DNA were tested as competitors of the biotinylated ^{5m}C dsDNA:TET binding interaction. **a** Competition for NgTet1 binding. **b** Competition for $TET2_{CD\Delta LCI}$ binding. **c** Counter screen for assay interference by double-stranded competitor DNA using a biotinylated His_6 peptide. **d** Tabulated competition pIC_{50} and IC_{50} values. Conditions: **a** 100 nM NgTet1 (50 nM ^{5m}C dsDNA), **b** 12.5 nM $TET2_{CD\Delta LCI}$ (12.5 nM ^{5m}C dsDNA), **c** 1.6 nM biotinylated His_6 peptide. Each assay was supplemented with 50 µM sodium L-ascorbate, 5 µM $(NH_4)_2Fe(SO_4)_2$, and 5 µM disodium NOG. Data shown as the mean of three to four experimental replicates (n = 3–4; mean ± SD); tabulated data reported as the mean of two independent replicates (n = 2).

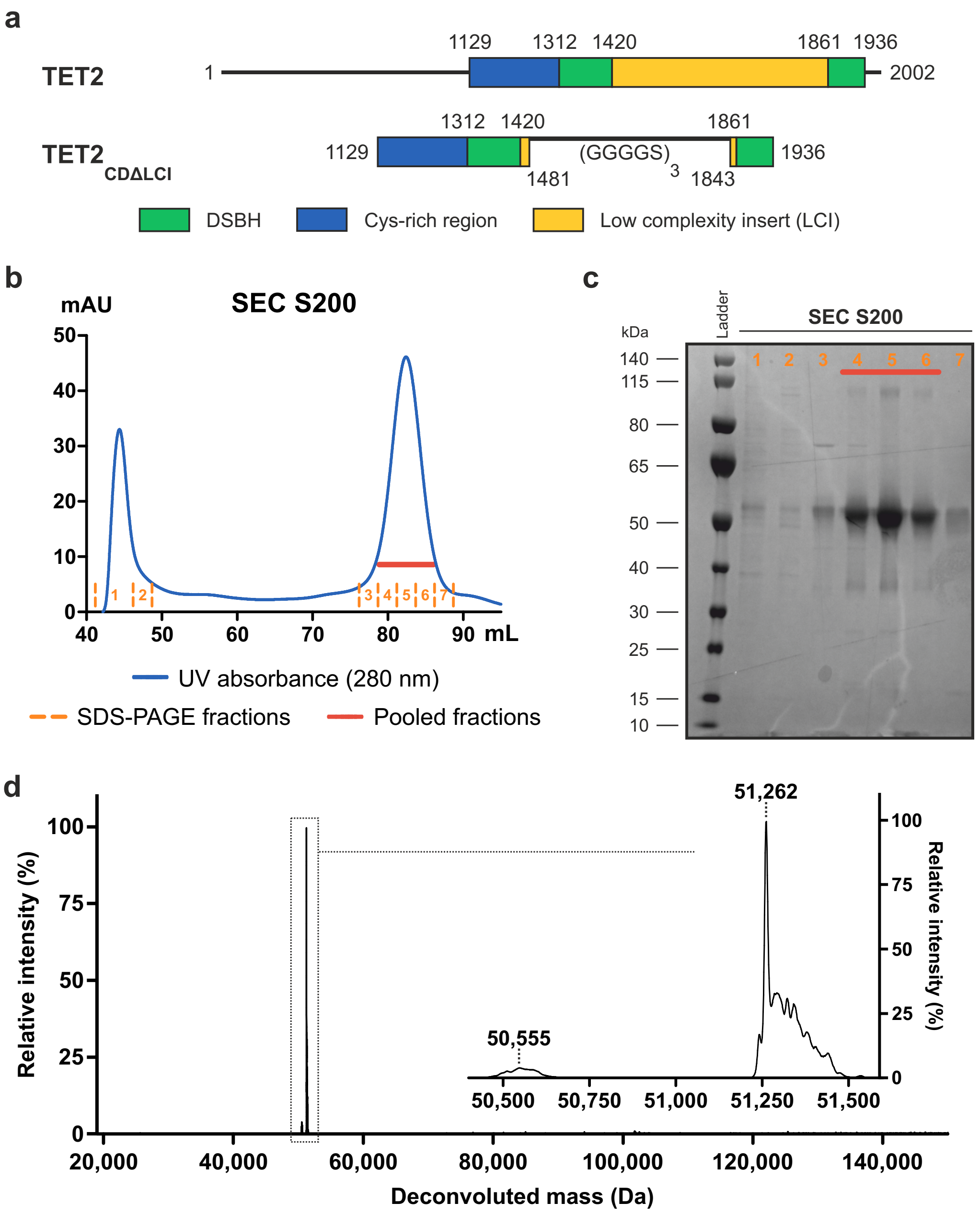

Fig. S6 Recombinant production of TET2$_{CD\Delta LCI}$ in *E. coli* BL21(DE3)-R3-pRARE2 strain. **a** Domain architecture of full-length TET2 and recombinant TET2 catalytic domain construct with an insertion-deletion in the low complexity insert region (TET2$_{CD\Delta LCI}$) used in this work[3]. **b** 280 nm elution profile of Superdex® 200 size exclusion chromatography step (SEC S200). **c** SDS-PAGE analysis of SEC S200 fractions; red bar indicates pooled fractions. **d** LC-MS analysis of purified TET2$_{CD\Delta LCI}$ (theoretical mass: 51,260 Da, observed mass: 51,262 Da). DSBH, double-stranded β-helix.

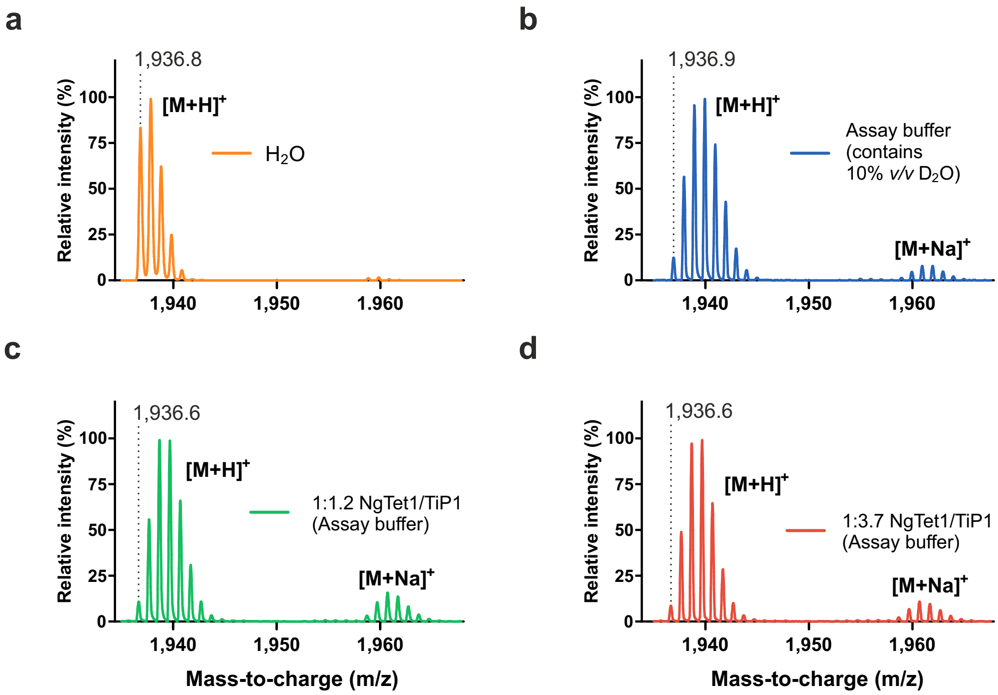

Fig. S7 MALDI-TOF MS analysis of cyclic peptide TiP1 stability under DNA substrate-free ^{1}H NMR assay conditions. a TiP1 in H_2O. **b** TiP1 in assay buffer (50 mM Tris-d_{11}, pH 7.5, 10% *v/v* D_2O). **c**, **d** TiP1 in assay buffer following incubation with NgTet1 and cofactors for 1 h at room temperature in the absence of substrate ^{5m}C DNA (1:1.2 and 1:3.7 ratios of NgTet1/TiP1, respectively). The observed change in TiP1 isotopic pattern (**b**, **c**, **d**) was attributed to peptide hydrogen-deuterium exchange enabled by the presence of 10% *v/v* D_2O in solution[4]. No TiP1 oxidation was detected following incubation with NgTet1, demonstrating that the observed increase in 2OG decarboxylase activity was not a result of oxidation product formation following TiP1 recognition as substrate by NgTet1.

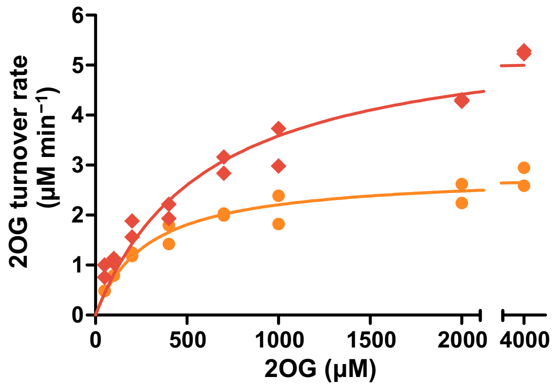

TiP1	K_M^{app} (2OG, µM)	V_{max}^{app} (µM min^{-1})	k_{cat}^{app} (min^{-1})	k_{cat}^{app} / K_M^{app} (min^{-1} µM^{-1})
● 0 equiv.	284	2.83	0.57	2.01×10^{-3}
◆ 1.2 equiv.	606	5.76	1.15	1.90×10^{-3}

Fig. S8 Michaelis-Menten enzyme kinetics plots and corresponding parameters for 2OG decarboxylation by NgTet1 with and without cyclic peptide TiP1 determined by ^{1}H NMR assay in the absence of ^{5m}C DNA substrate. TiP1 was added at a final concentration of 6.17 µM (1:1.2 ratio of NgTet1/TiP1). Data plotted from two independent replicates (n = 2); data reported as the mean of two independent replicates (n = 2).

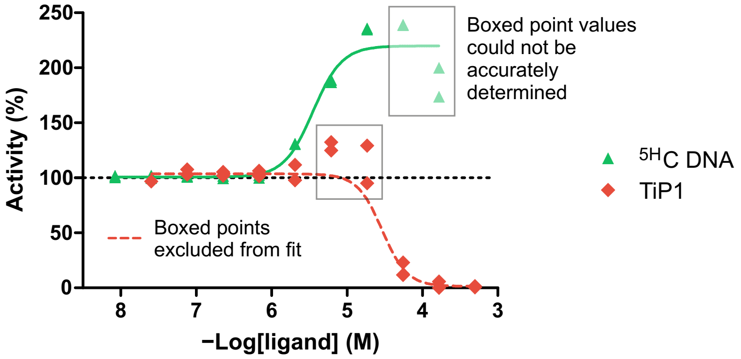

Fig. S9 Uncoupled NgTet1 2OG turnover stimulation dose-response curves for ^{5H}C DNA and TiP1 using ^{1}H NMR in the absence of ^{5m}C DNA substrate. ^{5H}C DNA produced a stronger allosteric activation effect on 2OG decarboxylation than TiP1 (188% and 137% activity relative to control at 6.17 μM ^{5H}C DNA and cyclic peptide TiP1, respectively). Values for the top two concentrations of ^{5H}C DNA (boxed, shaded) could not be accurately determined due to ^{1}H NMR signal overlap. Dose-response curve fitting for TiP1 was carried out excluding the two anomalous data points (boxed). Activity was normalised against reactions containing vehicle only (100% activity). Data is shown as the mean of 2 independent replicates (n = 2; mean ± SD).

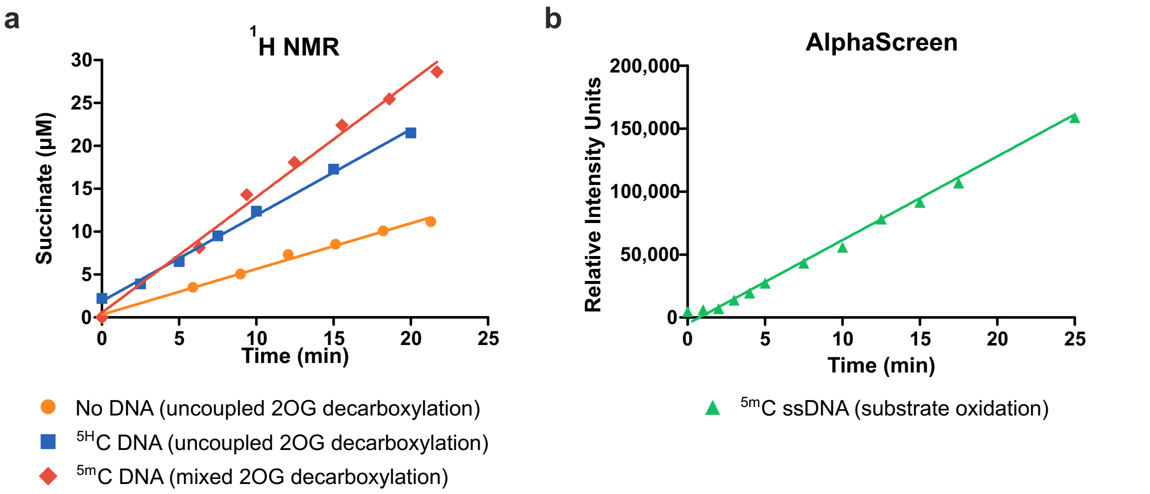

Fig. S10 Determination of linear NgTet1 enzymatic activity windows for inhibitor characterisation. a ^{1}H NMR succinate detection time courses with no DNA, ^{5H}C DNA (double-stranded self-complementary 5'-ACC ACC GGT GGT-3'), and ^{5m}C DNA (double-stranded self-complementary 5'-ACC AC^{5m}C GGT GGT-3'). **b** AlphaScreen ^{5hm}C detection time course using ^{5m}C ssDNA (single-stranded 5'-[Biotin]-TCG GAT GTT GTG GGT CAG ^{5m}CGC ATG ATA GTG TA-3'). All reactions exhibited excellent linearity within the following time intervals: [No DNA, ^{1}H NMR] R^2 = 0.992 (21.3 min); [^{5H}C DNA, ^{1}H NMR] R^2 = 0.996 (20.0 min); [^{5m}C DNA, ^{1}H NMR] R^2 = 0.994 (18.6 min); [^{5m}C ssDNA, AlphaScreen] R^2 = 0.994 (25.0 min). Representative time courses shown. ^{1}H NMR reaction conditions: 5 μM NgTet1, 2 mM sodium L-ascorbate, 100 μM $(NH_4)_2Fe(SO_4)_2$, 50 μM disodium 2OG, and 1% *v/v* DMSO-d_6 in 50 mM Tris-d_{11} [pH 7.5], 10% *v/v* D_2O. DNA was added to a final concentration of 20 μM of double-stranded DNA. AlphaScreen reaction conditions: 400 nM NgTet1, 10 nM ^{5m}C ssDNA, 100 μM sodium L-ascorbate, 10 μM $(NH_4)_2Fe(SO_4)_2$, 10 μM disodium 2OG, and 1% *v/v* DMSO in assay buffer (50 mM HEPES [pH 7.3], 150 mM NaCl, 0.1% *v/v* BSA, 0.01% *v/v* Tween® 20).

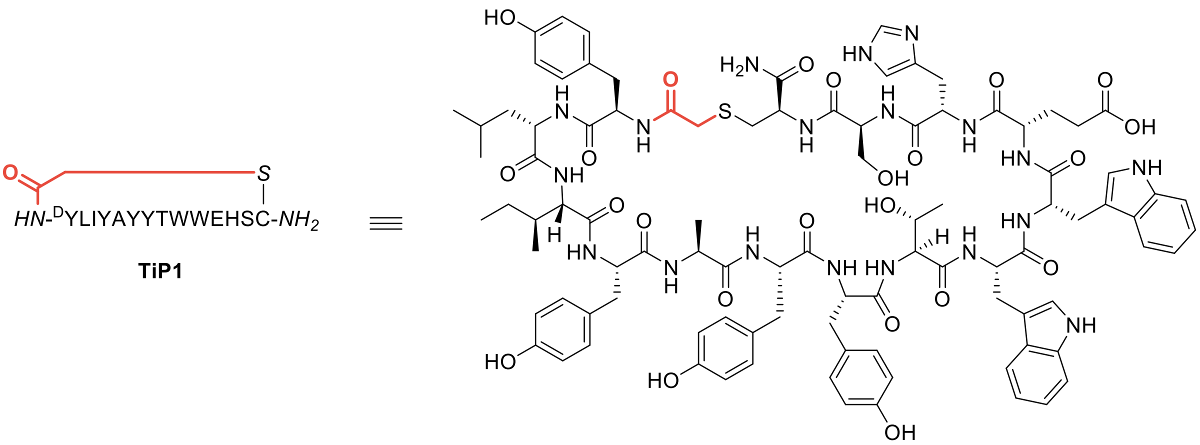

Fig. S11 Schematic representation (left) and chemical structure (right) of cyclic peptide TiP1. Letters representing individual atoms are italicised in the schematic representation (left) to distinguish from single-letter amino acid codes. Single letter code ^{D}Y refers to D-tyrosine.

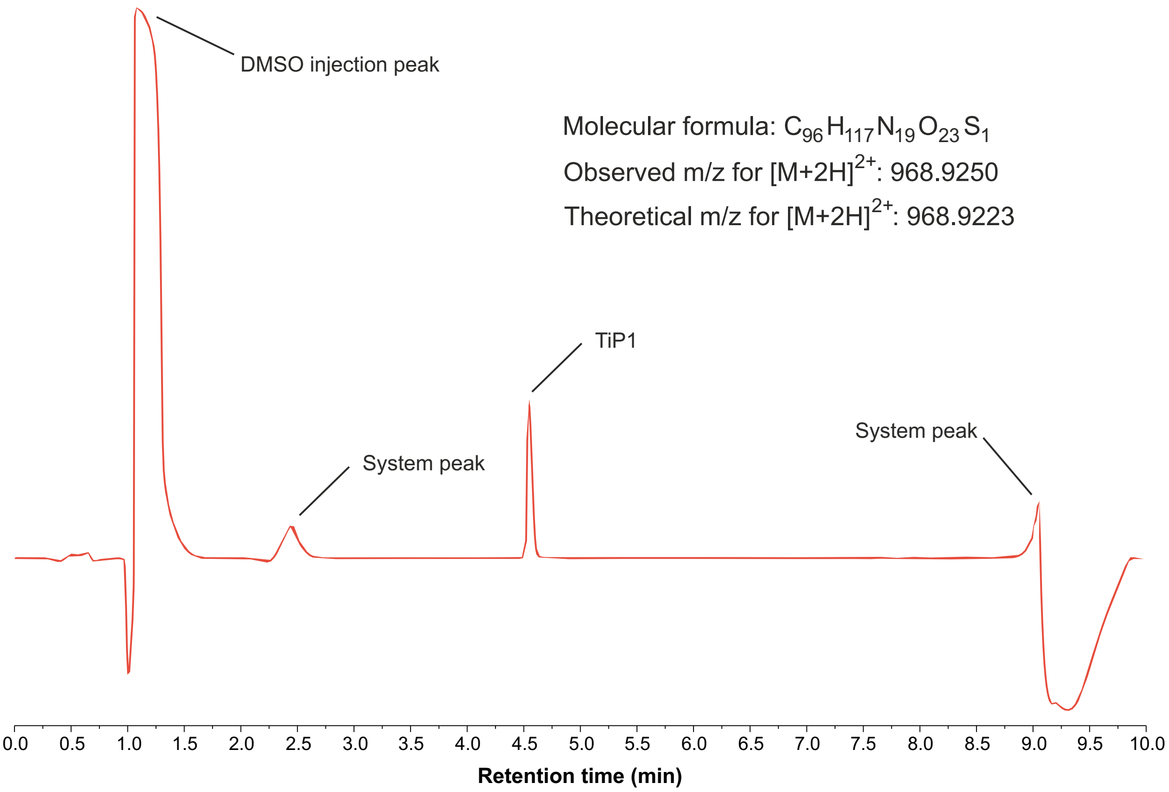

Fig. S12 LC-UV and HR-MS analysis of cyclic peptide TiP1. Peptide purity was shown to be >99% by LC-UV (220 nm wavelength); peptide identity was confirmed with a mass error of 2.8 ppm by HR-MS.

Supplementary References

(1) Hirel, P. H.; Schmitter, M. J.; Dessen, P.; Fayat, G.; Blanquet, S. Extent of N-Terminal Methionine Excision from Escherichia Coli Proteins Is Governed by the Side-Chain Length of the Penultimate Amino Acid. *Proc. Natl. Acad. Sci.* **1989**, *86* (21), 8247–8251. https://doi.org/10.1073/pnas.86.21.8247.

(2) Geoghegan, K. F.; Dixon, H. B. F.; Rosner, P. J.; Hoth, L. R.; Lanzetti, A. J.; Borzilleri, K. A.; Marr, E. S.; Pezzullo, L. H.; Martin, L. B.; Lemotte, P. K.; McColl, A. S.; Kamath, A. V.; Stroh, J. G. Spontaneous α-N-6-Phosphogluconoylation of a "His Tag" in Escherichia Coli: The Cause of Extra Mass of 258 or 178 Da in Fusion Proteins. *Anal. Biochem.* **1999**, *267* (1), 169–184. https://doi.org/10.1006/abio.1998.2990.

(3) Hu, L.; Li, Z.; Cheng, J.; Rao, Q.; Gong, W.; Liu, M.; Shi, Y. G.; Zhu, J.; Wang, P.; Xu, Y. Crystal Structure of TET2-DNA Complex: Insight into TET-Mediated 5mC Oxidation. *Cell* **2013**, *155* (7), 1545–1555. https://doi.org/10.1016/j.cell.2013.11.020.

(4) Pirrone, G. F.; Wang, H.; Canfield, N.; Chin, A. S.; Rhodes, T. A.; Makarov, A. A. Use of MALDI-MS Combined with Differential Hydrogen-Deuterium Exchange for Semiautomated Protein Global Conformational Screening. *Anal. Chem.* **2017**, *89* (16), 8351–8357. https://doi.org/10.1021/acs.analchem.7b01590.
