# Supplementary material for: Unravelling 2-oxoglutarate turnover and substrate oxidation dynamics in 5-methylcytosine-oxidising TET enzymes
Source: Commun Chem. 2024 Dec 20;7:305. doi: 10.1038/s42004-024-01382-1 (PMC11662004; doi:10.1038/s42004-024-01382-1)
Supplement: Supplementary file 2 — Description of Additional Supplementary Files [file 42004_2024_1382_MOESM2_ESM.pdf]

Description of Additional Supplementary Files

File name: Supplementary Data 1

Description: Numerical source data for graphs and charts
